# Simple and Sensitive Analysis of Blonanserin and Blonanserin C in Human Plasma by Liquid Chromatography Tandem Mass Spectrometry and Its Application

**DOI:** 10.1155/2014/629343

**Published:** 2014-02-11

**Authors:** Yunliang Zheng, Xingjiang Hu, Jian Liu, Guolan Wu, Huili Zhou, Meixiang Zhu, You Zhai, Lihua Wu, Jianzhong ShenTu

**Affiliations:** Research Center for Clinical Pharmacy, State Key Laboratory for Diagnosis and Treatment of Infectious Diseases, First Affiliated Hospital, Zhejiang University, Hangzhou 310003, China

## Abstract

A highly sensitive, simple, and rapid liquid chromatography tandem mass spectrometry method to simultaneously determine blonanserin and blonanserin C in human plasma with AD-5332 as internal standard (IS) was established. A simple direct protein precipitation method was used for the sample pretreatment, and chromatographic separation was performed on a Waters XBridge C_8_ (4.6 × 150 mm, 3.5 **μ**m) column. The mobile phase consists of a mixture of 10 mM ammonium formate and 0.1% formic acid in water (A) and 0.1% formic acid in methanol (B). To quantify blonanserin, blonanserin C, and IS, multiple reaction monitoring (MRM) was performed in positive ESI mode. The calibration curve was linear in the concentration range of 0.012–5.78 ng*·*mL^−1^ for blonanserin and 0.023–11.57 ng*·*mL^−1^ for blonanserin C (*r* ^2^ > 0.9990). The intra- and interday precision of three quality control (QC) levels in plasma were less than 7.5%. Finally, the current simple, sensitive, and accurate LC-MS/MS method was successfully applied to investigate the pharmacokinetics of blonanserin and blonanserin C in healthy Chinese volunteers.

## 1. Introduction

Blonanserin (AD-5423) is a novel oral atypical antipsychotic agent with relatively selective potent activity of blocking dopamine D2 and serotonin 5-HT2A receptors, which is expected to have lower incidence of adverse events than other antipsychotics [1–3]. Clinical trials have demonstrated that blonanserin is well tolerated and effective in the treatment of both positive and negative symptoms of schizophrenia, without extrapyramidal symptoms [4–6]. Blonanserin is rapidly absorbed orally and extensively metabolized and is metabolized mainly by the cytochrome P450 enzyme CYP3A4 according to data from in vitro studies [7, 8]. Several metabolites (N-oxide form, deethyl form, and 7- and 8-hydroxylated form) have been identified or estimated in in vivo and in vitro studies; among them, N-diethyl blonanserin (blonanserin C) is its major metabolite [7–12]. Some data indicated that blonanserin C could reduce the extrapyramidal symptoms (EPS) of blonanserin, which contributes at least partly to the atypical nature of blonanserin with low EPS liability [3].

Blonanserin has been sold in Japan and Korea and will be commercialized in China and some other countries in the world, indicated for the treatment of schizophrenia. With the development of clinical trials and clinical use on blonanserin throughout the world, it is necessary to establish a simple, sensitive, and accurate method for the quantification of blonanserin and its metabolite in human plasma. High-performance liquid chromatography (HPLC) with fluorescence detection (HPLC-FLD) [9], GC-MS [13], HPLC-MS/MS [14, 15], and UPLC-MS/MS [16, 17] have been reported for the determination of blonanserin in human plasma. However, they all need a complicated and expensive sample pretreatment method, either solid-phase extraction (SPE) or liquid-liquid extraction (LLE), for the purifying of plasma samples, so as to get a low limit of quantification (LOQ).

In this paper, a sensitive HPLC-MS/MS method for the simultaneous determination of blonanserin and its major metabolite (blonanserin C) in human plasma was established, which has a simple direct protein precipitation method for the sample pretreatment and has a higher extraction recovery (>85%) and a lower LOQ than the existing studies. It has been successfully applied to a pharmacokinetic study in healthy Chinese volunteers.

## 2. Experimental

### 2.1. Chemicals and Reagents

**Figure 1:**
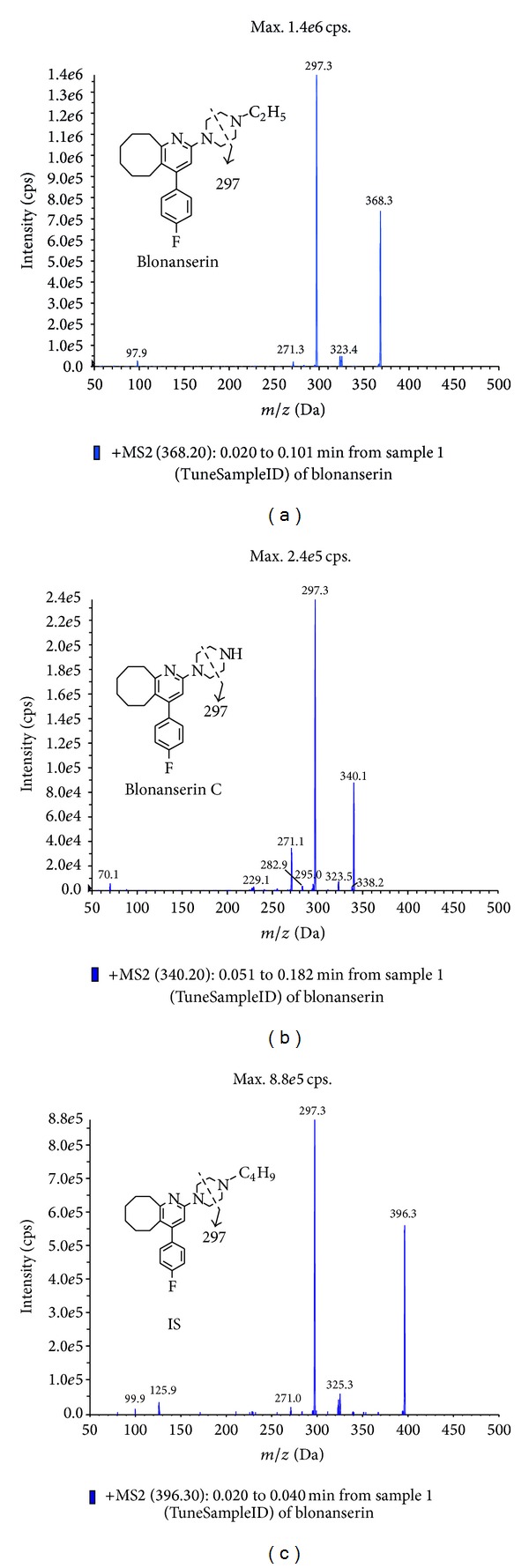
The structures and MS^2^ spectrums of blonanserin (a), blonanserin C (b), and IS (c).

Blonanserin (*batch number 20110124, purity 99.8%*), blonanserin C (*batch number 20120509, purity 99.8%*), and AD-5332 (internal standard, IS) (*batch number 20121101, purity 99.6%*) were supplied by the Medical Research Institute Co., Ltd., Jiangsu Stockhausen, China. Their chemical structures are shown in Figure 1. HPLC grade methanol and acetonitrile were purchased from Merck KGaA Company (Darmstadt, Germany). HPLC grade ammonium formate and formic acid were purchased from Sigma (St. Louis, MO, USA). Water was purified using a Milli-Q system (Milford, MA, USA). Blank human plasma (without blonanserin) was obtained from the Blood Center of Zhejiang Province (Hangzhou, China).

### 2.2. Instruments

A Spark Holland Symbiosis HPLC system (Spark Holland, Emmen, Netherlands) coupled to an API 4000 triple-quadrupole mass spectrometer (AB Sciex, Ontario, Canada) via a electrospray ionization (ESI) interface was used for separation and detection. Data acquisition was performed with Analyst 1.5.2 software (AB Sciex).

### 2.3. LC-MS/MS Conditions

Separation was performed using a Waters XBridge C_8_ (4.6 × 150 mm, 3.5 *μ*m) column. A guard column (XDB C_8_; 4.6 × 12.5 mm, 5 *μ*m) was placed prior to the analytical column. A binary mobile phase consisting of a mixture of 10 mM ammonium formate and 0.1% formic acid in water (A) and 0.1% formic acid in methanol (B) was used at a flow rate of 0.6 mL·min^−1^. The temperature of column was 35°C, and the injection volume was 10 *μ*L. Gradient elution was performed according to the following elution program: 0 min, 20% A/80% B; 2 min, 10% A/90% B; 4 min 30 sec, 10% A/90% B; 4 min 35 sec, 5% A/95% B; 5 min 30 sec, 5% A/95% B; 5 min 35 sec, 20% A/80% B.

All measurements were carried out with mass spectrometer operated under the positive ESI mode. The multiple reaction monitoring (MRM) transitions (Q1 and Q3) and declustering potential (DP), collision energy (CE), and collision exit potential (CXP) of blonanserin, blonanserin C, and IS are shown in Table 1. Other parameters: collision gas (CAD), curtain gas (CUR), ion source gas1 (GS1), and ion source gas2 (GS2) were 6, 20, 65, and 65 psi, respectively; dwell time was 200 ms; ion spray voltage and temperature were 4000 V and 450°C, respectively.

### 2.4. Preparation of Standard Solution and Quality Control (QC) Samples

Accurately weighed solid portions of standards (11.56 mg, 7.71 mg, and 5.25 mg for blonanserin, blonanserin C, and IS, resp.) were dissolved in methanol to prepare stock solutions separately as follows: 1.156 mg·mL^−1^ for blonanserin, 0.771 mg·mL^−1^ for blonanserin C, and 0.525 mg·mL^−1^ for IS. Working solutions used for spiking plasma were all freshly prepared by diluting the stock solutions with methanol to appropriate concentrations.

Calibration standards were prepared by freshly spiking the appropriate working solution into blank pooled plasma to prepare concentrations of 0.012–5.78 ng·mL^−1^ for blonanserin and 0.023–11.57 ng·mL^−1^ for blonanserin C and processed as described in the preparation of sample solution (Section 2.5). The QC samples (at three concentration levels, refer to Tables 2-3) used for the recovery study, intraday and interday accuracy, precision, and stability study, were prepared in the same way as the preparation of calibration standards and then stored at −20°C until analysis.

### 2.5. Sample Extraction Procedures

After frozen human plasma samples were thawed at ambient temperature and adequately vortexed, a total of 200 *μ*L aliquot plasma sample was added to 20 *μ*L of methanol (supplementary volume) and 20 *μ*L IS (5.25 ng·mL^−1^) solution. After a thorough vortex mixing for 30 s, and mixtures were precipitated with 400 *μ*L acetonitrile, vortex-mixed for 30 s, centrifuged at 13000 rpm for 5 min, and then 10 *μ*L of supernatant was injected into the HPLC-MS/MS system.

### 2.6. Method Validation

The selectivity, linearity, sensitivity, precision, and extraction recovery of the method and the stability of blonanserin and blonanserin C in plasma samples were assessed and investigated. To evaluate selectivity, drug-free plasma samples from 6 individuals were analyzed to check for the presence of any interfering peaks at the elution times of blonanserin, blonanserin C, and IS. The calibration curves were constructed using 7 standards ranging in concentration from 0.012 to 5.78 ng·mL^−1^ for blonanserin and 0.023 to 11.57 ng·mL^−1^ for blonanserin C. Calibration curves were constructed from the peak-area ratios of each analyte to IS versus plasma concentrations using a 1/*x* weighted least squares linear regression model.

The intraday and interday precisions were evaluated by assessing QC samples at the following concentrations (*n* = 6): LLOQ (0.012 ng·mL^−1^ and 0.023 ng·mL^−1^), low (0.023 ng·mL^−1^ and 0.046 ng·mL^−1^), medium (0.23 ng·mL^−1^ and 0.46 ng·mL^−1^), and high (4.62 ng·mL^−1^ and 9.25 ng·mL^−1^) for blonanserin and blonanserin C, respectively. The relative standard deviation (RSD %) was calculated.

The extraction recoveries of blonanserin and blonanserin C for the low-, medium-, and high-concentration QC samples were determined by comparing the results of spiked plasma solutions to unspiked plasma. Stability tests involved leaving the untreated plasma sample at ambient temperature for 3 h without light, placing the treated plasma sample in an autosampler for 24 h, three freeze-thaw cycles from −80°C to room temperature, and storing for 60 days at −80°C. Stability analysis was performed using three aliquots of each QC sample at three different concentrations.

### 2.7. Application of the Assay

The validated method was used to determine the concentrations of blonanserin and blonanserin C in plasma samples 0–72 h after oral administration of blonanserin tablets to 6 healthy Chinese volunteers (3 females and 3 males, 20 to 24 years old) in a pharmacokinetic study, which has been approved by the ethics committee. The volunteers were given three dosages with single oral administration: low, 4 mg/volunteer, middle, 8 mg/volunteer, and high, 12 mg/volunteer, respectively. The study was conducted from low dosage to high dosage, with one week washout period between two dosages. Heparinized blood samples (5 mL) were collected at the following times: before (0.0 h) and at 0.5, 1.0, 1.5, 2.0, 3.0, 4.0, 6.0, 8.0, 12.0, 16.0, 24.0, 36.0, 48.0, and 72.0 h after dosing. The blood samples were centrifuged at 4000 rpm for 10 min, and plasma samples were separated and stored at −80°C until analyzed.

## 3. Results and Discussion

### 3.1. Optimization of LC-MS/MS Condition

Mass spectrometer parameters were derived from analyte infusion experiments using a syringe pump (typical concentration was 115 ng·mL^−1^, 77 ng·mL^−1^, and 104 ng·mL^−1^ in HPLC solvent B for blonanserin, blonanserin C, and IS, resp.). ESI source with positive or negative ionization has been tested for the determination, and the results revealed that blonanserin, blonanserin C, and IS were more sensitive in positive ionization mode. Based on the Q1 and product ion scan (Figure 1) three MRM transitions were chosen. The DP, CE, and CXP for each transition and ion source parameters were also optimized: the final parameters are shown in part “2.4. LC-MS/MS conditions” and Table 1.

**Figure 2:**
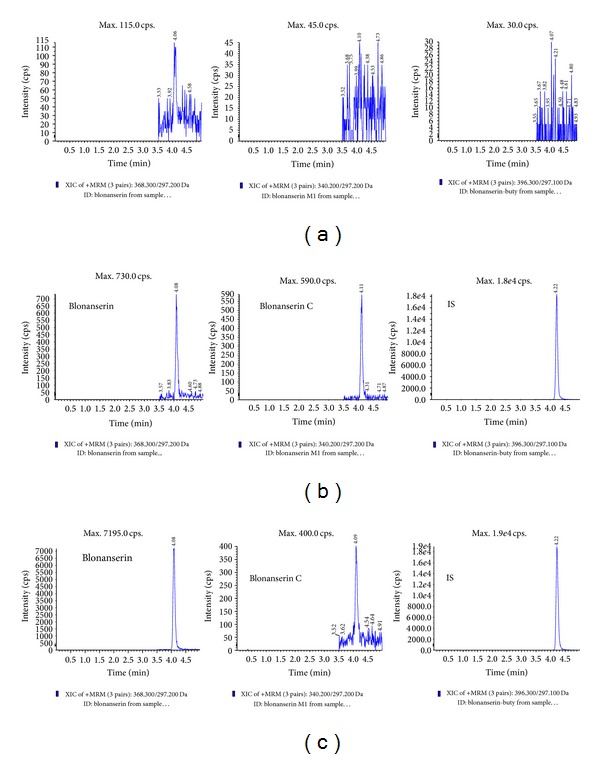
MRM chromatograms of blonanserin, blonanserin C, and IS obtained from human plasma samples: (a) blank plasma; (b) blank plasma spiked with standard solution (LLOQ); (c) plasma sample from a healthy subject 1.5 h after oral administration of 8 mg blonanserin tablet.

To optimize chromatography, stationary phase, composition of mobile phase, and column temperature were investigated in the LC domain. Waters XBridge C_8_ (4.6 × 150 mm, 3.5 *μ*m) column was chosen in the present study for its good peak symmetry. Different mobile phases (acetonitrile-water and methanol water with different concentrations of modifiers: formic acid and ammonium formate) were examined to obtain efficient chromatography and relatively short run time for the analytes and IS. It was found that the addition of ammonium formate and formic acid could remarkably improve the peak symmetry and sensitivity of blonanserin, blonanserin C, and IS. When 0.1% formic acid in methanol was used as the organic phase, the sensitivity of blonanserin and blonanserin C was further improved. Therefore, the mobile phase was selected as 0.1% formic acid in methanol mixture of 10 mM ammonium formate and 0.1% formic acid in water to achieve higher sensitivity and less interference of other components in the plasma. The retention time for blonanserin, blonanserin C, and IS was 4.08, 4.09, and 4.22 min, respectively (Figure 2), and the total chromatographic run time was 5.5 min.

### 3.2. Method Validation

#### 3.2.1. Selectivity

The typical MRM chromatograms of mixed blank plasma from six blonanserin- and blonanserin C-free individuals, a spiked plasma sample with the two analytes at LLOQ and IS and a plasma sample from a healthy volunteer 1.5 h after an oral administration, are shown in Figure 2. The results indicated that there was no apparent endogenous interference for the determination of blonanserin and blonanserin C. We have also injected and analyzed the single standard of blonanserin or blonanserin C crossly with only one of the MRM transitions and got no peek in the two injections, so there was no crosstalk effect between blonanserin and blonanserin C in this method.

#### 3.2.2. Linearity of Calibration Curves and Limit of Quantification (LOQ)

The standard calibration curves for spiked human plasma containing blonanserin and blonanserin C were linear over the range of 0.012~5.78 ng·mL^−1^ and 0.023~11.57 ng·mL^−1^, respectively. Good linearity was observed for the analyte using a weighted (1/*x*) least squares linear regression analysis with coefficient of determination *r* ^2^ > 0.9990. Typical equations for the calibration curve were as follows: *Y* = (2.04 ± 0.18)*X* + (0.0121 ± 0.0039) and *Y* = (1.00 ± 0.05)*X* + (0.0057 ± 0.0032)  (*n* = 6) for blonanserin and blonanserin C, respectively, where *X* represents the plasma concentration of blonanserin or blonanserin C (ng/mL) and *Y* represents the ratio of blonanserin or blonanserin C peak area to that of IS.

The LOQ under the optimized conditions was 0.012 ng/mL and 0.023 ng/mL for blonanserin and blonanserin C, which were judged from the fact that the precision was less than 20% and the *S*/*N* ratios were higher than 10. The LOQ of this is sufficient for the pharmacokinetic study of blonanserin and blonanserin C following an oral administration.

#### 3.2.3. Extraction Recovery (ER), Matrix Effects (ME), Precision, and Accuracy

The RE and ME (%) of blonanserin and blonanserin C in human plasma of this method were determined in three concentration levels (six replicates per level). Results summarized in Table 2 were calculated through the following formula (1):

(1) $$\begin{array}{c} \text{ME}\;\left(\%\right)=\frac{B}{A}\times 100\\ \text{ER}\;\left(\%\right)=\frac{C}{B}\times 100. \end{array}$$

“*A*” is the peak area obtained in neat solution standards; “*B*” is the corresponding peak area for standards spiked *after* extraction into plasma extracts; “*C*” is the peak area for standards spiked *before* extraction.

QC samples at three concentration levels were calculated over three validation runs (once a day). Six replicates of each QC level were determined in each run. Table 2 summarizes the intraday and interday precision and accuracy for blonanserin and blonanserin C.

The extraction recovery (>85%) and precision (<7.2%) for blonanserin and blonanserin C were all better than in references [9, 15, 17], which demonstrated the good accuracy and repeatability of this described method.

#### 3.2.4. Stability

The results of stability studies are shown in Table 3, which indicated that blonanserin and blonanserin C were stable under the four different conditions.

### 3.3. Application

**Figure 3:**
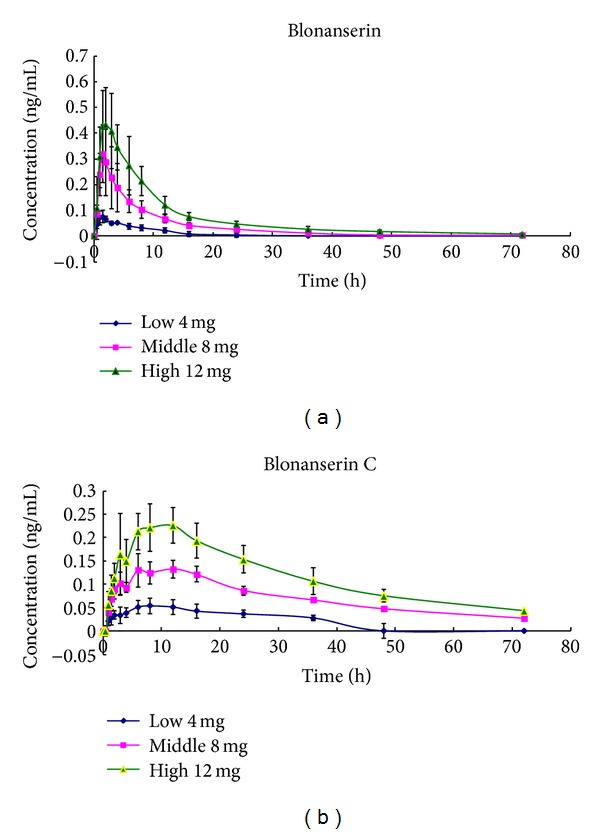
Mean plasma concentration-time profile of blonanserin and blonanserin C after oral administration of blonanserin tablets (low, 4 mg, middle, 8 mg, and high, 12 mg) to 6 healthy subjects.

The validated method was proved to be simple and sensitive enough to determine the concentration up to 72 h after dose of blonanserin tablets to 6 healthy Chinese volunteers. The precision and accuracy of the calibration and QC samples were well within the acceptable limits [18]. The pharmacokinetic parameters were calculated by drug and statistics (DAS) software, version 2.1.1 (Shanghai, China). The mean ± S.D. (*n* = 6) plasma concentration versus time profiles for blonanserin and blonanserin C is depicted in Figure 3, and the main pharmacokinetic parameters for blonanserin and blonanserin C are summarized in Table 4.

The results from the pharmacokinetic trial in humans showed that after oral administration of the drug, the pharmacokinetics of blonanserin and blonanserin C within the used dosage range (4, 8, and 12 mg/volunteer) were in accordance with linear pharmacokinetic characteristics. Figure 3 (blonanserin C) showed that the concentrations of point seven (collected at 4 h after administration) were lower than that of point six (collected at 3 h after administration), which may be caused by the eating of lunch at 11:30 a.m. (about 3.5 h after administration). In order to clarify the specific mechanisms of the phenomenon, further studies need to be carried out.

## 4. Conclusion

A simple and sensitivity direct protein precipitation-LC/MS/MS method for the quantification of blonanserin and blonanserin C in human plasma was developed. Method validation has been demonstrated by a variety of tests for selectivity, linearity, sensitivity, precision, recovery, and stability. This method is attractive for the pharmacokinetic analysis of blonanserin and blonanserin C, because of its simple sample processing steps, high analytical sensitivity, and accuracy.

## Figures and Tables

**Table 1:** ESI^+^-MS/MS parameters of the parent and daughter ions (*m/z*) and DP, CE, and CXP of blonanserin, blonanserin C, and IS.

Analytes	Q1 (*m*/*z*)	Q3 (*m*/*z*)	DP (V)	CE (V)	CXP (V)
Blonanserin	368.3	297.2	100	36	20
Blonanserin C	340.2	297.2	120	40	8
IS	396.3	297.1	100	36	8

**Table 2:** Extraction recovery, matrix effect, precision, and accuracy of the developed method (*n* = 6).

Analytes	QC Conc. (ng·mL^−1^)	Extraction recovery (%)	Matrix effect (%)	Intraday (R.S.D, %)	Interday (R.S.D, %)	Accuracy (%)
Blonanserin	0.023	85.04 ± 9.84	110.6 ± 3.45	6.41	7.19	98.71
	0.23	95.21 ± 5.49	112.8 ± 4.17	2.96	4.98	101.2
	4.62	89.95 ± 2.26	105.3 ± 4.35	4.05	3.69	97.06

Blonanserin C	0.046	91.40 ± 6.41	105.9 ± 4.28	4.34	5.41	98.49
	0.46	86.15 ± 11.82	110.5 ± 13.39	3.50	5.37	101.7
	9.25	89.00 ± 2.58	96.22 ± 4.57	4.05	4.52	97.76

**Table 3:** Stability of the three analytes in rats plasma under different storage conditions (*n* = 5).

Analytes	QC Conc. (ng·mL^−1^)	Short term (5 h, 25°C)	Autosampler 25°C for 24 h	Three freeze-thaw cycles	Long term (60 days, −20°C)
Blonanserin	0.023	4.52	1.01	−12.77	2.94
	0.23	4.52	−2.60	−0.95	8.13
	4.62	0.42	−2.54	−4.70	−1.31

Blonanserin C	0.046	−7.12	−2.16	−10.79	−2.08
	0.46	2.25	0.07	−8.20	8.08
	9.25	−3.34	−0.36	−9.46	−1.68

Stability values are represented as R.E. (%) = [Found Conc. − QC Conc.]/QC Conc. × 100%.

**Table 4:** Mean pharmacokinetic parameters for blonanserin and blonanserin C (*n* = 6).

Compound	Parameters	4 mg	8 mg	12 mg
Blonanserin	*T*_max⁡_ (h)	2.37 ± 1.43	1.88 ± 0.75	2.50 ± 1.23
	*C*_max ⁡_ (ng·mL^−1^)	0.08 ± 0.02	0.32 ± 0.11	0.49 ± 0.12
	*T*_1/2_ (h)	6.42 ± 2.58	15.29 ± 13.06	16.46 ± 3.70
	AUC_0–*t*_ (ng·h·mL^−1^)	0.56 ± 0.16	2.56 ± 1.10	4.90 ± 1.32
	AUC_0–*∞*_ (ng·h·mL^−1^)	0.70 ± 0.22	2.88 ± 1.32	5.15 ± 1.33

Blonanserin C	*T*_max⁡_ (h)	8.50 ± 2.52	9.50 ± 3.00	7.25 ± 3.78
	*C*_max⁡_ (ng·mL^−1^)	0.05 ± 0.02	0.15 ± 0.02	0.24 ± 0.04
	*T*_1/2_ (h)	28.52 ± 7.50	26.24 ± 3.93	26.25 ± 2.90
	AUC_0–*t*_ (ng·h·mL^−1^)	1.50 ± 0.53	5.11 ± 0.33	8.39 ± 1.48
	AUC_0–*∞*_ (ng·h·mL^−1^)	2.48 ± 0.50	6.10 ± 0.28	10.04 ± 1.49
